# Identification and Functional Verification of CITED2 Gene Promoter Region in Patients with Patent Ductus Arteriosus

**DOI:** 10.3390/ijms242216204

**Published:** 2023-11-11

**Authors:** Zhuo Chen, Huan-Xin Chen, Hai-Tao Hou, Xiu-Yun Yin, Qin Yang, Guo-Wei He

**Affiliations:** The Institute of Cardiovascular Diseases & Department Cardiovascular Surgery, TEDA International Cardiovascular Hospital, Tianjin University & Chinese Academy of Medical Sciences, Tianjin 300457, China; chloe_981126@tju.edu.cn (Z.C.); chenhx@tedaich.com (H.-X.C.); houht@tedaich.com (H.-T.H.); 20209063@stu.wnmc.edu.cn (X.-Y.Y.); yangq@tedaich.com (Q.Y.)

**Keywords:** patent ductus arteriosus, CITED2, promoter, genetic, gene variant

## Abstract

Patent ductus arteriosus (PDA) is a common congenital heart disease. CITED2 plays an important role in the development of the heart, and genetic variants in its coding region are significantly associated with cardiac malformations. However, the role of variants in the promoter region of CITED2 in the development of PDA remains unclear. We extracted the peripheral blood of 646 subjects (including 353 PDA patients and 293 unrelated healthy controls) for sequencing. We identified 13 promoter variants of the CITED2 gene (including 2 novel heterozygous variants). Of the 13 variants, 10 were found only in PDA patients. In mouse cardiomyocytes (HL-1) and rat cardiac myocytes (RCM), the transcriptional activity of the CITED2 gene promoter was significantly changed by the variants (*p* < 0.05). The results of the experiments of electrophoretic mobility indicated that these variants may affect the transcription of the CITED2 gene by influencing the binding ability of transcription factors. These results, combined with the JASPAR database analysis, showed that the destruction/production of transcription factor binding sites due to the variants in the promoter region of the CITED2 gene may directly or indirectly affect the binding ability of transcription factors. Our results suggest for the first time that variants at the CITED2 promoter region may cause low expression of CITED2 protein related to the formation of PDA.

## 1. Introduction

Patent ductus arteriosus (PDA) is a common type of congenital heart disease (CHD) in children, accounting for 15% of the total incidence of CHD [1]. PDA occurs in term infants at a rate of 3 to 8 per 10,000 live births [2] and is estimated to be as high as 30% in very-low-birth-weight infants (less than 1500 g at birth) [3]. PDA primarily affects the heart and often causes recurrent infections of the lungs. Diagnosis is usually made by cardiopulmonary auscultation and echocardiography. The PDA may be closed by intervention or surgery, and symptomatic treatment is combined with non-steroidal anti-inflammatory drugs and other drugs [4]. Generally, patients can live a normal life after surgery, but for those with severe pulmonary hypertension, the postoperative prognosis is still poor [5,6]. At present, the etiology of PDA is unclear, but related to genetic, maternal, and environmental factors [1,7,8].

CITED2 (CBP/p300 interacting transactivators with Glu/Asp rich carboxy-terminal domain2) is located on chromosome 6q23 and is an important member of the CITED family of transcriptional auxiliary factors. CITED2 is a non-DNA-binding transcriptional coactivator that plays a key role in the development of embryos and extraembryonic tissues, such as cardiac morphogenesis [9]. It was reported that CITED2 plays a role in the left–right patterning through the Nodal-PITX2C pathway [10]. Further, CITED2 knockout mice die in utero and display various cardiac malformations with multiple organs affected [9,11]. At present, studies have shown that the occurrence of various CHDs may be related to CITED2 gene variants [12,13,14]. However, most of them are concentrated in the coding region.

The promoter can be recognized by RNA polymerase and can interact with transcription factors that determine the initiation of transcription during ribonucleic acid synthesis, and then control which protein the cell starts to produce [15]. DNA sequence variants in gene promoter regions may be associated with changes in gene expression levels that may lead to disease [16]. Based on our previous studies on the pathogenic mechanism of CITED2 variants in CHD, this study investigated possible variants in the promoter region of CITED2 in PDA patients. Further, the functional changes related to the variants were examined at the cellular level.

## 2. Results

### 2.1. Variant Analysis of CITED2 Gene Promoter Region

**Figure 1:**
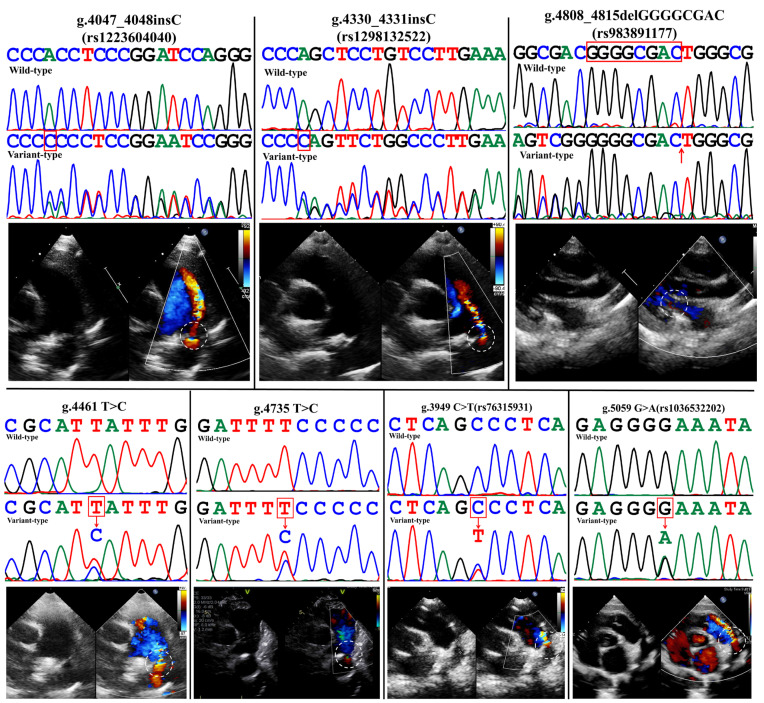
Sanger sequencing results from the 3’ end to the 5’ end and the relative transcriptional activity of CITED2 gene promoter. Sanger sequencing map of CITED2 gene promoter variation and echocardiography of PDA patients. The variants only discovered in CITED2 promoter of PDA patients and verified by cell function in this study are shown as g.3949C > T(rs76315931), g.4047_4048insC(rs1223604040), g.4330_4331insC(rs1298132522), g.4461T > C, g.4735T > C, g.4808_4815delGGGGCGAC(rs983891177), and g.5059G > A(rs1308068829). Top panel: wild type. Bottom panel: hybrid variant, followed by Doppler echocardiography screenshot of the corresponding PDA patient. The red arrow in the panel of g.4808_4815delGGGGCGAC(rs983891177) indicates the position of the missing fragment “GGGGCGAC”. The DNAMAN comparison results for variant rs983891177 are presented in the Supplementary Material placed as Figure S2. The white dashed circle marks the position of the PDA. rs number: a systematic identifier applied to human genomics to locate individual positions in genotype analysis, also known as SNPs. The two variants not labeled with rs numbers are novel variants discovered for the first time in this study.

A total of 13 variants were found in 646 subjects. In the PDA group, two new variants (g.4461T > C and g.4735T > C) were found, which were not included in the NCBI dbSNP database (https://www.ncbi.nlm.nih.gov/snp/, accessed on 6 December 2022). Eight single-nucleotide polymorphisms (SNPs) include two inserts (g.4047_4048insC(rs1223604040), g.4330_4331insC(rs1298132522), g.4808_4815delGGGGCGAC(rs983891177), g.3949C > T(rs76315931), g.4935C > T(rs111470468), g.5027C > T(rs112831934), g.5059G > A(rs1308068829), and g.5108C > T(rs995827210)). Among these SNPs, g.5027C > T(rs112831934) was found in four PDA patients. The other three common variants (g.4285T > G(rs12333191), g.4357G > A(rs76757432), and g.5122C > A(rs570422697)) were also found in the control group and excluded from the next study. Table 1 lists these variants and their allele frequencies from the gnomAD database (http://gnomad-sg.org/, accessed on 6 December 2022). In addition, variants g.4935C > T(rs111470468), g.5027C > T(rs112831934), and g.5108C > T(rs995827210) have been shown to significantly alter CITED2 promoter activity in our previous studies [17,18], which were only validated in the present study in rat cardiac myocytes (RCMs). The rest of the validation was not repeated and their sequencing maps are not shown in Figure 1.

### 2.2. Effect of CITED2 Promoter Variant on Dual-Luciferase Assay

**Figure 2:**
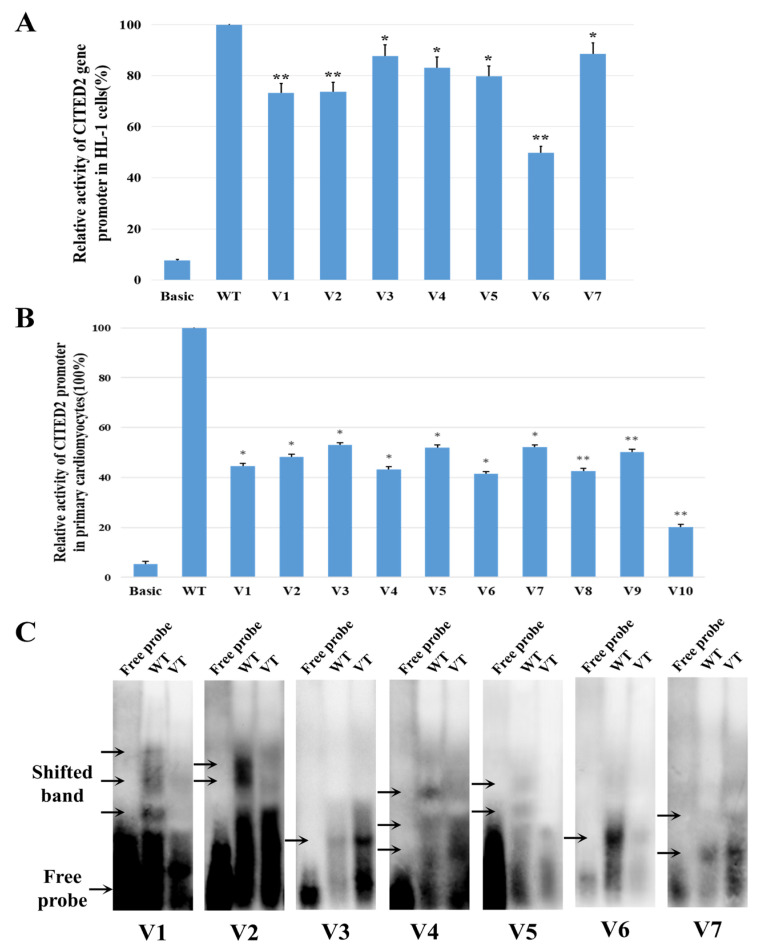
Results of functional validation of variants in the promoter region of CITED2 at the cellular level and the possible consequences of variants in cardiac development. (**A**) Relative transcriptional activity of CITED2 gene promoter in HL-1 cells. The transcriptional activity of the wild-type CITED2 gene promoter was set to 100%, and the relative activity of the variant was calculated. The experiment was repeated three times independently. Basic, pGL3 empty vector; WT, wild type; V1, g.3949C > T(rs76315931); V2, g.4047_4048insC(rs1223604040); V3, g.4330_4331insC (rs1298132522); V4, g.4461T > C; V5, g.4735T > C; V6, g.4808_4815delGGGGCGAC(rs983891177); V7, g.5059G > A(rs1308068829). * *p* < 0.05; ** *p* < 0.01. (**B**) Relative transcriptional activity of CITED2 gene promoter in RCMs. The transcriptional activity of the wild-type CITED2 gene promoter was set to 100%, and the relative activity of the variant was calculated. The experiment was repeated three times independently. Basic, pGL3 empty vector; WT, wild type; V1, g.3949C > T(rs76315931); V2, g.4047_4048insC(rs1223604040); V3, g.4330_4331insC(rs1298132522); V4, g.4461T > C; V5, g.4735T > C; V6, g.4808_4815delGGGGCGAC(rs983891177); V7, g.5059G > A(rs1308068829); V8, g.4935C > T(rs111470468); V9, g.5108C > T(rs995827210); V10, g.5027C > T (rs112831934). * *p* < 0.05; ** *p* < 0.01. (**C**) Results of electrophoretic mobility shift analysis (EMSA). All variants affected binding capacity of the transcription factor. Free probes: marked on the bottom; arrows: affected binding of unknown transcription factors; WT: wild type; VT: variant.

The luciferase reporter gene expression vectors were constructed with the wild-type CITED2 gene promoter and variant promoter, respectively. The expression vector of the wild-type CITED2 gene promoter was named pGL3-WT. Variant expression vectors included V1: pGL3-3949T, V2: pGL3-4047_4048insC, V3: pGL3-4330_4331insC, V4: pGL3-4461C, V5: pGL3-4735C, V6: pGL3-4808_4815delGGGGCGAC, V7: pGL3-5059A, V8: pGL3-g.4935T, V9: pGL3-g.5027T, and V10: pGL3-g.5108T. The empty vector pGL3 was used as a negative control. Transfected mouse cardiomyocytes (HL-1) and rat cardiac myocytes (RCMs) were collected to measure dual fluorokinase activity. The transcriptional activity of the wild-type CITED2 gene promoter was set at 100%, and the relative transcriptional activities of the variants with the CITED2 promoter and the wild type were calculated separately. As shown in Figure 2A, luciferase expression was lower (*p* < 0.05) in HL-1 compared with the wild type for all variants, with similar findings and more pronounced downregulation (*p* < 0.05) in RCMs, as shown in Figure 2B.

### 2.3. Changes in Transcription Factor Binding Sites Are Affected by Genetic Variant

To determine whether genetic variants affect the putative binding sites of transcription factors (TFs), the CITED2 gene promoter (accessed by http://jaspar.genereg.net/, on 12 December 2022) was analyzed using the JASPAR database. The results showed that these five SNPs (g.3949C > T(rs76315931), g.4047_4048insC(rs122360404040), g.4330_4331insC(rs1298132522), g.4808_4815delGGGGCGAC(rs983891177), g.5059G > A(rs1308068829)) may destroy or produce potential transcription factor binding sites (TFBs). Table 2 summarizes the analysis data.

In order to further study whether the variant affects the binding ability of transcription factors, EMSA (electrophoretic mobility shift analysis) experiments were conducted using wild-type or variant biotin-labeled probes. As expected, seven significant variants (g.4047_4048insC(rs1223604040), g.4330_4331insC(rs1298132522), g.4461T > C, g.4735T > C, g.4808_4815delGGGGCGAC(rs983891177), g.3949C > T(rs76315931), g.5059G > A(rs1308068829)) affected CITED2 gene transcription by affecting the binding capacity of the transcription factors (Figure 2C). The original EMSA map is shown in Figure S1. As shown in the figure, although bands are also present in most of the variant-type lanes, the different shades of the bands indicate that the binding ability of the variant type to the transcription factors is different from the wild type.

## 3. Discussion

This study identifies for the first time variants in the CITED2 promoter in patients with PDA compared with controls. In this study, (1) we found a total of 13 variants in this region, and 10 of these variants were found only in PDA patients; (2) all the variants found in PDA patients significantly altered the expression of the CITED2 gene; and (3) these variants played a role in the development of PDA by affecting the binding ability of the promoter to the TF or by altering the TFBs on the promoter.

The human CITED2 gene consists of two exons and two introns. Variants in the coding region of the CITED2 gene have been reported in various human CHDs, including VSD, ASD, TOF, etc. [12,14,19]. However, there are no reports on the role of variants in the promoter region of CITED genes in PDA.

In this study, the sequences of the CITED2 promoter and its flanking region were detected in 646 samples. Two new sequence variants (g.4461T > C and g.4735T > C) and eight SNPs (g.4047_4048insC(rs1223604040), g.4330_4331insC(rs1298132522), g.3949C > T(rs76315931), g.4808_4815delGGGGCGAC(rs983891177), g.4935C > T(rs111470468), g.5027C > T (rs112831934), g.5059G > A(rs1308068829), and g.5108C > T(rs995827210)) were identified only in PDA patients. Importantly, these variants significantly affect the transcriptional activity of the CITED2 gene promoter by changing TF binding. Interestingly, the variants g.4935C > T(rs111470468), g.5027C > T (rs112831934), and g.5108C > T(rs995827210) found in PDA patients in this study were also found in different types of CHD (isolated TOF and ASD) in our previous studies [17,18]. The effect of g.5027C > T on the cellular function was studied in different organelles (HEK-293 and HL-1) and reported [17,18], and similar results were obtained in the RCM in the present study. An increasing number of studies have demonstrated that many heart defects are not necessarily due to overall changes in genomic content but are usually caused by alterations in gene expression due to certain specific variants of genes [19]. This is supported by our studies mentioned above on the genetic aspects of CHD which found that the same pathogenic variants cause different phenotypes, indicating the importance of these variants in the development of CHD. Further cell function experiments confirmed this conjecture. These experiments showed similar results in RCM and HL-1 cells in this study and in HEK-293 cells in the previous study [17,18], although the reduction in the relative activity was more significant in the RCM than HL-1 cells. This may reflect the fact that primary cardiomyocytes might more closely resemble the cellular state in vivo than cell lines. In future experiments, primary cardiomyocytes could be more frequently used. These results demonstrated that these variants affected the expression of CITED2 in different types of cells. This fact reveals the importance of these two variants in the pathogenesis of CHD.

CHD is usually associated with genes necessary for heart development, and in patients with non-syndromic CHD, it is likely to be involved in transcription factors (TBX1, FOG2, GATA4, NKX2.5, etc.) or signaling pathways (FOXH1, VEGF, NODAL, NOTCH1, etc.) that may disrupt cardiac morphogenesis [19]. CITED2 was also found to play critical roles in expression regulation and early and late cardiac development [20]. Transcription factors are proteins that control gene transcription by binding to specific DNA sequences. Transcription factors can act as activators or repressors by activating or disabling the ability of ribonucleic acid polymerases to bind to specific genes. The results of EMSA showed altered (aggravated or attenuated) lane protein blocking bands in the variant compared with the wild type, suggesting that the CITED2 promoter region is affected by the variant, and that the variant enhances or attenuates the ability of the CITED2 promoter to bind to transcription factors. Different from the dual fluorokinase reporter analysis, variants in EMSA altered the ability to bind to other transcription factors on the CITED2 promoter, not necessarily consistent with functional changes in CITED2 itself. There are not only synergistic but also antagonistic effects between transcription factors during cardiac development; therefore, some of the CITED2 promoter variants cause overexpression of transcription factors but downregulation in the luciferase assay.

In view of this, the variants in the CITED2 promoter may result in the production/destruction of TFB, and then affect the expression level of CITED2. In the prediction results of the JASPAR database shown in Table 2, CITED2 acts as a bridge connecting the neural crest transcription factor TFAP2 and the p300/CBP transcriptional coactivation complex, and the interaction of the three is critical for the normal development of the cardiac neural crest [10]. E2F1/4 is involved in the regulation of CITED2 expression in neurons after stroke-related injury [21]. SP1, ETV4, STAT3, and YBX1 are also involved in the occurrence of different diseases with CITED2 by cooperating/repressing transcription factors or signaling pathways [22,23,24,25].

This study has certain limitations. The genotype–phenotype associations with the discovered variants in PDA patients need to be further confirmed. In particular, the role of these variants in the development of the heart and CHD may require experiments with transgenic animal models.

Although the present study demonstrated the pathological role of CITED2 promoter variants, it did not use whole-genome sequencing of the DNA of the PDA patients, and this would have missed the possible interaction of the variants of the CITED2 gene with other genes. Therefore, we do not suggest that the variants are the only factors in the development of PDA. CHD often develops with the effect of multiple genetic variations interacting with each other. The variants found in this study may have interacted with other genes to produce PDA in the clinical setting.

## 4. Methods

### 4.1. Participants

**Figure 3:**
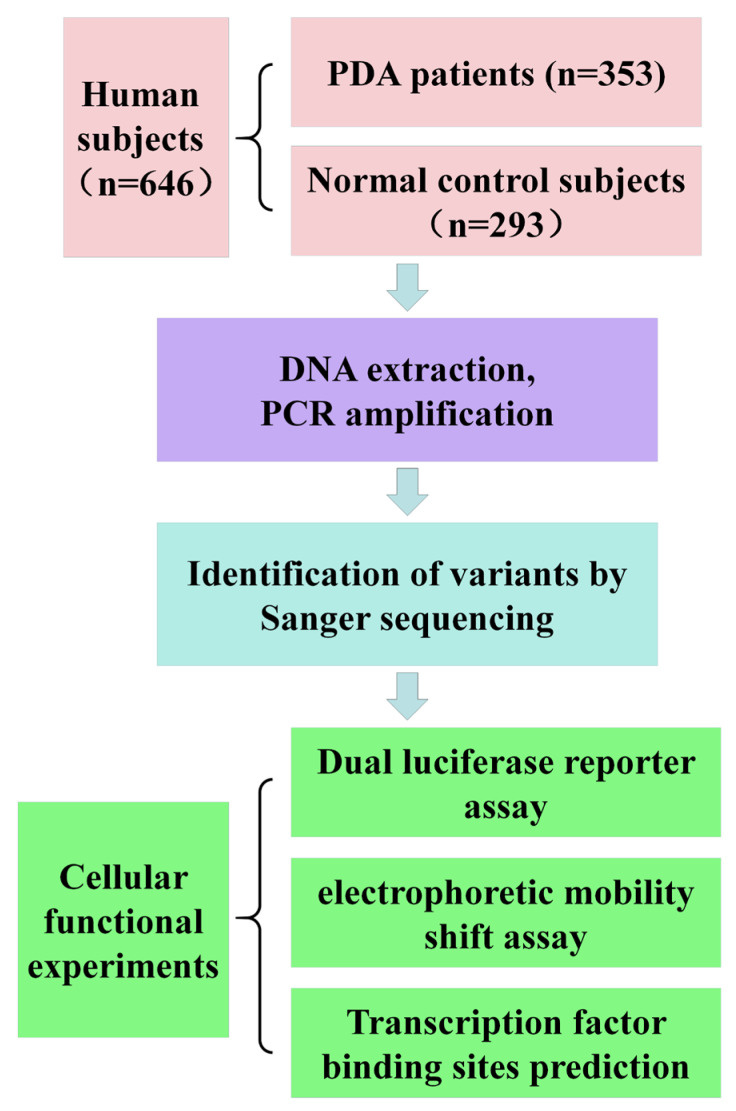
Study flow diagram: schematic diagram of the dual fluorophore enzyme report and results of the variant screening. Flow chart of the study. The study recruited 646 subjects. The study procedures included: sequence analysis, cell function experiments, electrophoretic mobility shift analysis, and bioinformatics.

A total of 646 samples from a Chinese Han population were collected in this study, including from 353 PDA patients and 293 healthy controls of the same race and age. There were 226 females and 127 males diagnosed with isolated PDA, with the average age of 4 years. All CHD patients with other CHDs were excluded from this study. The diagnosis was based on clinical manifestations, echocardiograms, and CT scans when necessary. The diagnosis was confirmed in all patients during corrective heart surgery at TEDA International Cardiovascular Hospital of Tianjin University in China. The control group was selected from routine health checkups or CHD screening programs. All control subjects were confirmed free of heart disease and other familial genetic diseases. In addition, none of the subjects in the control group had other major diseases, determined by physical examination and echocardiography. Figure 3 shows the flow diagram of the study.

The study followed the principles of the Declaration of Helsinki and was approved by the Institutional Review Board of TEDA International Cardiovascular Hospital (Clinical Research Ethics Review Number: 2021-0715-4). Written informed consent was obtained from the parents or guardians of all subjects.

### 4.2. Genomic DNA Extraction and Sequence Analysis

Peripheral blood samples were collected from all patients and controls as we previously reported. Genomic DNA was extracted from the peripheral blood of each subject using a Blood Genomic DNA Extraction Kit (Tiangen, Beijing, China) following the manufacturer’s instructions. The DNA samples were then stored at −20 °C. Primers were designed according to the CITED2 reference sequence (NCBI: NG_016169.1). The CITED2 promoter sequence (1418 bp, from −1197 bp to +220 bp) and its flanking regions were obtained by PCR, and the products were sequenced directly. The primers required for PCR amplification and Sanger sequencing are listed in Table S1. The sequencing results were compared with the reference sequence by Chromas 2.6.5 and DNAMAN 6.0 software to identify the variant.

### 4.3. Site-Directed Mutagenesis and Plasmid Construction

In a previous experiment [26], we amplified the wild-type fragment of the CITED2 gene promoter containing KpnI and BglII terminal restriction sites. The wild type was subcloned into the KpnI/BglII site upstream of the firefly luciferase reporter gene plasmid (pGL3-basic) by restriction enzyme digestion to construct an expression vector, and Sanger sequencing was performed. The reporter plasmid pGL3 Basic-CITED2 (WT) with firefly luciferase reporter gene was obtained. CITED2 variant plasmids were constructed using a QuickMutation™ Site-Directed Mutagenesis Kit (Biyotime, Shanghai, China). All constructed plasmids were then sequenced to verify the absence of nucleotide errors during plasmid construction. The constructed plasmids were purified and transfected into Escherichia coli DH5a, and the positive clones were selected for reproduction. A large number of fresh bacterial solution was obtained, and the plasmids were extracted using a Plasmid Mini Preparation Kit (Biyotime, Shanghai, China).

### 4.4. Cell Culture, Transfection, and Dual-Luciferase Assays

HL-1 cells were resuscitated at 37 °C in 5% CO_2_ and transfected with Lipo2000 (Invitrogen, Waltham, MA, USA) after 24 hours of passaging culture. Rat hearts were removed on the second day of life, and RCMs were isolated and cultured [27]. Cells were transfected after 72 h of incubation at 37 °C with 5% CO_2_. The Renilla luciferase reporter plasmid (PRL-SV40) was used as an internal control to standardize the transfection efficiency. An empty pGL3 base vector was used as a negative control. Plasmids containing pGL3-CITED2 (wild type or variant) or empty vector were co-transfected with the Renilla luciferase control plasmid. At 36 to 48 h after transfection, cells were washed with phosphate-buffered saline (PBS), collected, and completely lysed. Luciferase activity was then measured using a dual-luciferase reporter assay system (Thermo Scientific Fluoroskan FL, Waltham, MA, USA). The transcriptional activity of the CITED2 gene promoter was evaluated by measuring the ratio of firefly luciferase activity to Renilla luciferase activity. The relative activity of the CITED2 gene promoter and the variant was calculated using the wild-type CITED2 gene promoter activity as 100%. Experiments were repeated three times independently.

### 4.5. Transcription Factor Binding Site Prediction

JASPAR is an annotated, high-quality, matrix-based open-access database for mapping transcription factor binding sites in multicellular eukaryotes. We used the JASPAR database (https://jaspar.genereg.net/, accessed on 10 February 2023) to further study whether the variants in the promoter region of CITED2 will affect TFBs. Bioinformatics analysis using the JASPAR database showed that by comparing the predicted results of VT (variant) and WT, we can determine whether the variant destroys or produces potential TF binding sites (see JASPAR database for details). We set the relative profile score threshold to 85%.

### 4.6. Electrophoretic Mobility Shift Assay

Nuclear proteins were extracted from cells by using nuclear protein extraction kits (Beyotime, Shanghai, China). The oligonucleotide (30 bp) was labeled with biotin as a probe. Biotinylated double-stranded oligonucleotides (with or without variants) were synthesized. These were then used as labeled probes. After incubation for 20 min at room temperature, EMSA was performed using the chemiluminescence EMSA kit (Beyotime, Shanghai, China). Electrophoresis was carried out on a 4% polyacrylamide gel at 90 V for 1 h, and the membranes were transferred at 330 mA. After cross-linking the nylon membrane with ultraviolet light, the signal was detected by chemiluminescence.

### 4.7. Statistical Analysis

IBM SPSS 23.0 software was used for statistical analysis. Continuous variables were presented as mean ± SEM (standard error). Student *t*-test was used for comparison of quantitative data and chi-square for frequency of variants. The significance level was set as *p* < 0.05.

## 5. Conclusions

In this study, we found several genetic variants in the CITED2 gene promoter region in PDA patients. These variants may change the functional expression level of CITED2 by affecting TFBs, thus promoting the development of PDA. These data lay a foundation for further study on the impact of CITED2 gene variants on the occurrence of CHD and the related molecular mechanism.

## Figures and Tables

**Table 1:** Variants in the CITED2 gene promoter region discovered in PDA patients and control.

Variations	PDA ^a^	Controls ^a^	Position ^b^	Genotypes	Allele Frequency ^c^		*p*-Value
**Only Discovered in PDA Patients and Further Validated**					Total	East Asian	
g.3949 C > T(rs76315931)	1	0	−1052 bp	CT	A = 0.06612	A = 0.000	>0.9999 #
g.4461 T > C	1	0	−540 bp	TC	-	-	>0.9999 #
g.4735 T > C	1	0	−266 bp	TC	-	-	>0.9999 #
g.5059 G > A(rs1308068829)	1	0	+58 bp	GT	T = 0.00001314	T = 0.000	>0.9999 #
g.4047_4048insC (rs1223604040)	1	0	−954 bp_−953 bp	InsC	dupG = 0.00000	dupG = 0.00000	>0.9999 #
g.4330_4331insC (rs1298132522)	1	0	−671 bp_−670 bp	InsC	dupG = 0.00000	dupG = 0.00000	>0.9999 #
g.4808_4815delGGGGCGAC (rs983891177)	1	0	−193 bp_−186 bp	DelGGGGCGAC	-	-	>0.9999 #
g.4935C > T(rs111470468)	1	0	−66 bp	CT	A = 0.015720	A = 0.0003	>0.9999 #
g.5108 C > T(rs995827210)	1	0	+107 bp	CT	A = 0.000100	A = 0.0000	>0.9999 #
g.5027C > T (rs112831934)	4	0	+26 bp	CT	A = 0.0005846	A = 0.001541	0.1303
**Discovered in both PDA patients and control** **(No Further Validation)**							
g.4285T > G(rs12333191)	26	33	−716 bp	TG	C = 0.231281	C = 0.0467	0.7827
g.4357G > A(rs76757432)	3	16	−644 bp	GA	T = 0.094422	T = 0.0006	0.0084
g.5122C > A(rs570422697)	2	1	+121 bp	CA	T = 0.000029	T = 0.0013	0.5989

Abbreviations: -, not applicable; PDA, patent ductus arteriosus. ^a^ Allele frequency in groups. ^b^ Variants are located upstream (−) to the transcription start site; the position is 5001 (+1) of CITED2 (NG_016169.1). ^c^ GnomAD database. *p*-value was calculated by chi-square or Fisher’s exact test (#).

**Table 2:** Effects of the promoter region variants of the CITED2 gene on TFBs predicted by JASPAR database.

Variations	Binding Sites for Transcription Factors	
	Create	Disrupt
g.3949C > T (rs76315931)	BATF, ISL1, MZF1	BACH1, BACH2, FOSL2, ISL2, MAFK, NKX2-8, THAP1, ZNF264
g.4461T > C	GATA2, MSGN1, VAX1, VAX2	BARX1, E2F3, E2F3, ETV2, FOXL1, FOXP3, GSC, GSC2, GSX2, HOXB13, OTX1, OTX2, POU6F1, POU6F2, RAX, RHOXF2, SOX18
g.4735T > C	ERF, ETV2, SPDEF, ETV5, ETV7, FLI1, REL, RELA, RELB, TCF3, VEZF1	EGR1, NFATC2, PITX2, PITX3, SPI1, ZNF880
g.5059 G > A (rs1308068829)	E2F4, E2F6, ELK1::FOXI1, FOXC1, FOXC2, FOXD2, FOXP3, GATA3, NFATC2, NFATC4, PRDM1, STAT1::STAT2, THAP1, ZNF518A, ZNF98	CDX4, ETV6, ETV7, GATA3, HOXA10, HOXA7, HOXD13, HOXD9, KLF5, MYB, MZF1, NR2C2, RELB, SP5, VAX2, ZNF264, ZNF674
g.4047_4048insC (rs1223604040)	FOXL1, GL13, ZNF571	EGR1, GCM2, ZNF460
g.4330_4331insC (rs1298132522)	None	CTCFL, EGR1, TFAP2B, TFAP2C, THAP1
g.4808_4815delGGGGCGAC (rs983891177)	None	SOX18, SP1

## Data Availability

The individual SNP numbers are given in Table 2. The genetic variants described in this manuscript are available at https://www.ncbi.nlm.nih.gov/snp/ (accessed on 6 December 2022). Data supporting the findings of this study are available upon reasonable request from the corresponding authors.

## Supplementary Materials

The following supporting information can be downloaded at: https://www.mdpi.com/article/10.3390/ijms242216204/s1.
