# Supplementary material for: Identification and Functional Verification of CITED2 Gene Promoter Region in Patients with Patent Ductus Arteriosus
Source: Int J Mol Sci. 2023 Nov 11;24(22):16204. doi: 10.3390/ijms242216204 (PMC10671043; doi:10.3390/ijms242216204)
Supplement: Supplementary file 1 [file ijms-24-16204-s001.zip › ijms-2512815-supplementary.pdf]

Figure S1 Original image of electrophoretic migration analysis experiment.

Correspond to V1-V7 in Figure 4A

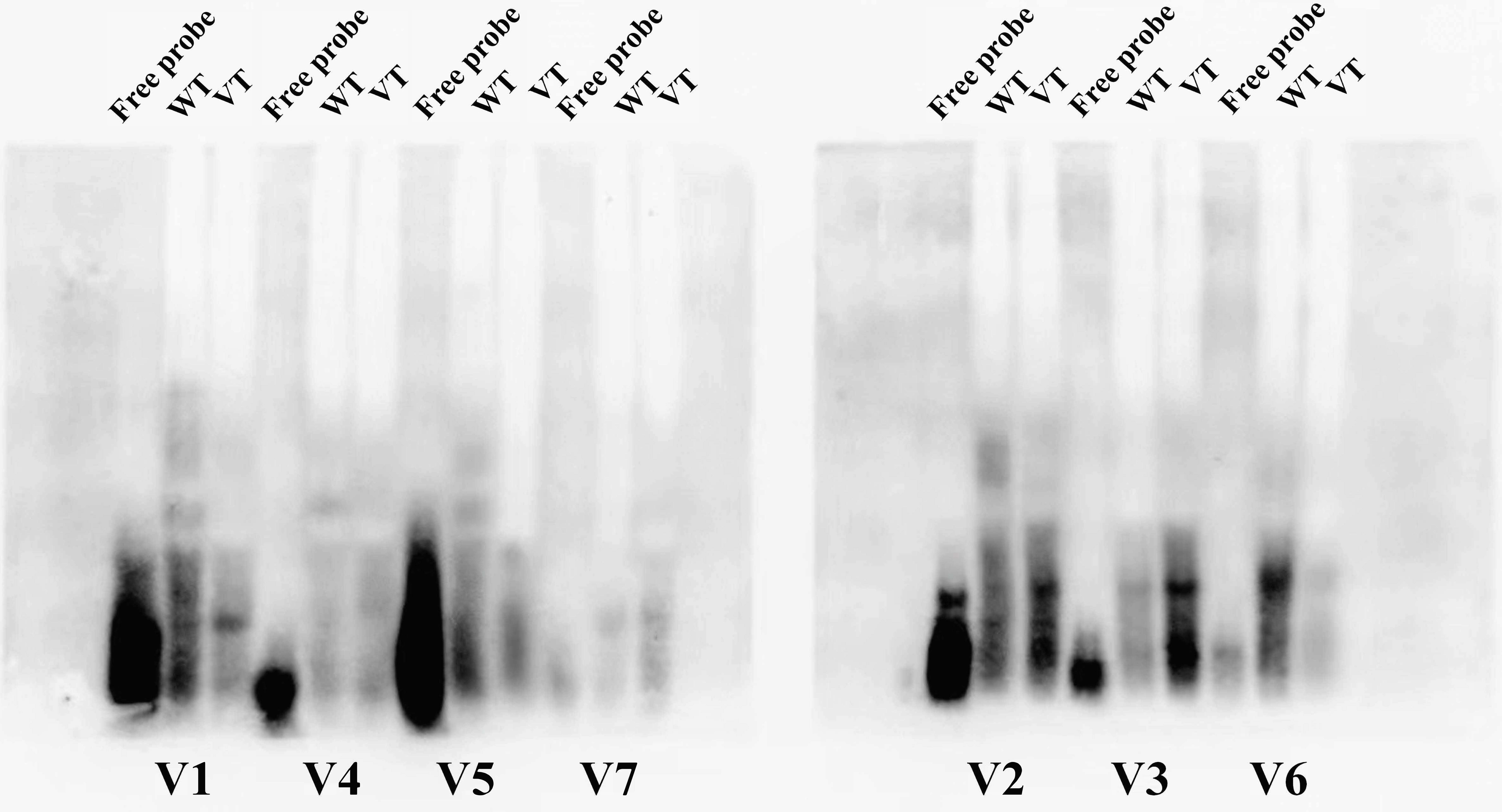

Figure S2 DNAMAN comparison results for variant rs983891177

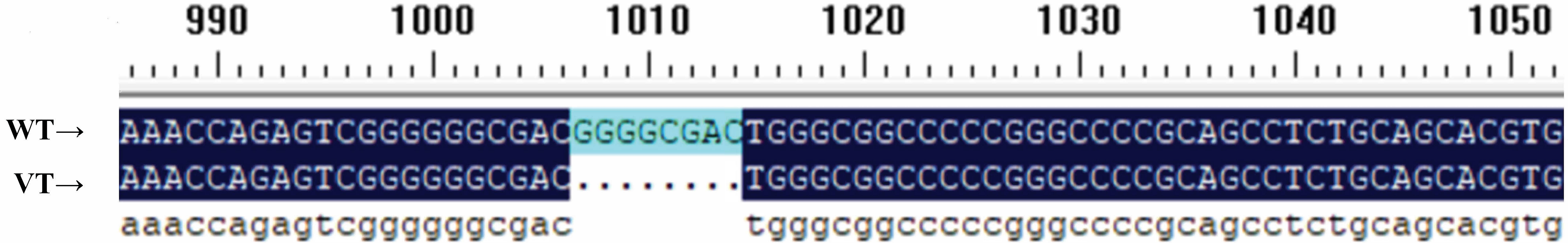

Table S1 Primers used in this study.

Primers	Sequences 5'-3'	Location	Position
**PCR and sequencing primers**			
CITED2-F1	5'-AAAGGAAGAGTCCCAGCCGT-3'	3804	-1197
CITED2-F2	5'-TTTCTGCTCCGAAGACCGAG-3'	5221	200
**Primers containing restriction sites**			
CITED2-KpnIa,b	5'-(KpnI)-**GG**GGTACCAAAGGAAGAGTCCCAGCCGT-3'	3804	-1197
	CCCAGCCGT-3'		
CITED3-BglIIa,b	5'-(BglII)-**CA**AGATCTTTTCTGCTCCGAAGACCGAG-3'	5221	200
	AGACCGAG-3'		
**The double-stranded biotinylated oligonucleotides for the EMSA**			
g.3949 C>T	5′-GCAAGAGCAGCCTTACTCAG(C/T)CCTCAAATTTCTTAATTAC-3′		
g.4461 T>C	5′-CAGGAAAGGGCGCAT(T/C)ATTTGTCCGGGTCTT-3′		
g.4735 T>C	5′-GGCAGGGGAGGATTT(T/C)CCCCCTGCCTCGG-3′		
g.5059 G>A	5′-GGGCAACGGAGGG(G/A)AAATAAAAGGGAACGGC-3′		
g.4047_4048ins	5′-CGTTTGTACACACCCCC(/C)ACCTCCCGGATCCAG-3′		
g.4330_4331insC	5′-CCTCCCCGCCCCC(/C)AGCTCCTGTCCTTGAAA-3′		
	g.4808_4815delGGGGCGAC	-	
